# Supplementary material for: Considerations on brain age predictions from repeatedly sampled data across time
Source: Brain Behav. 2023 Aug 16;13(10):e3219. doi: 10.1002/brb3.3219 (PMC10570486; doi:10.1002/brb3.3219)
Supplement: Supplementary file 1 — Supplement 1: Analyses on the travelling human phantom data set (data set 2) excluding repeat‐scans Supplement 2: Standardized quality control metrics at 1.5T for FTHP1 Supplement 3: Age, predicted age, brain age gap (BAG), and prediction error by cross‐sectional data set and field strength [file BRB3-13-e3219-s001.docx]

**Supplement for “Brain age predictions in longitudinal data reveal the importance of scan quality and field strength”**

Max Korbmacher^1,2,3^, Meng-Yun Wang^3,4^, Rune Eikeland^3,4^, Ralph Buchert^5^, Esten Leonardsen^2,6^, Lars T. Westlye^2,6^, Ivan I. Maximov^1,2,3^, Karsten Specht^3,4,7^

1 Department of Health and Functioning, Western Norway University of Applied Sciences, Bergen, Norway

2 Norwegian Centre for Mental Disorders Research (NORMENT), Oslo University Hospital & Institute of Clinical Medicine, University of Oslo, Oslo, Norway

3 Mohn Medical Imaging and Visualisation Center (MMIV), Bergen, Norway

4 Department of Biological and Medical Psychology, University of Bergen, Bergen, Norway

5 Department of Diagnostic and Interventional Radiology and Nuclear Medicine, University Medical Center Hamburg-Eppendorf, Martinistr. 52, 20246, Hamburg, Germany

6 Department of Psychology, University of Oslo, Oslo, Norway

7 Department of Education, UiT The Arctic University of Norway, Tromsø, Norway

**Supplement 1: Analyses on the travelling human phantom data set (data set 2) *excluding* repeat-scans**

This section describes the analyses conducted on data set 2, as described in the paper, but without including the N = 30 repeat scans in the analyses.

*Weak relationship between brain age and age (section follow-up)*

Similar to the first data set, in the second dataset, brain age was weakly correlated with chronological age *r_crude_* = 0.082, 95% CI [-0.003, 0.166], *p* = .060 in a subject scanned at multiple sites and varying scanning parameters such as field strength. Brain age predictions were more accurate at 3T (*r_partial_* = 0.357, 95% CI [0.208, 0.491], *p* < .001) than at 1.5T (*r_partial_* = 0.066, 95% CI [-0.036, 0.166], *p* = .204) when holding scanner site constant, as illustrated by a linear model predicting brain age from age (*β_std_* = 0.166, *p* <.001), field strength (*β_std_* = -0.456, *p* <.001), slice thickness (*β_std_* = 0.117, *p* =.002), and scanner (*β_std_* = -0.125, *p* =.002). Accordingly, holding field strength constant (ignoring other covariates), strengthened the relationship between age and brain age (*r_partial_* = 0.178, 95% CI [0.094, 0.260], *p* < .001).

*Scan quality: a possible reason for inaccurate brain age predictions?*

Similar to the analysis including repeat scans, none of the QC metrics were significant predictors of brain age (*p_Holm_* = 1), and of the acquisition paramters manufacturer, field strength and slice thickness, only field strength was a significant predictors of brain age (β_std_ = -1.156, *p*_Holm_ < .001), when using age as fixed effect and site ID, manufacturer, and slice thickness as random effects.

**Supplement 2: Standardized quality control metrics at 1.5T for FTHP1**


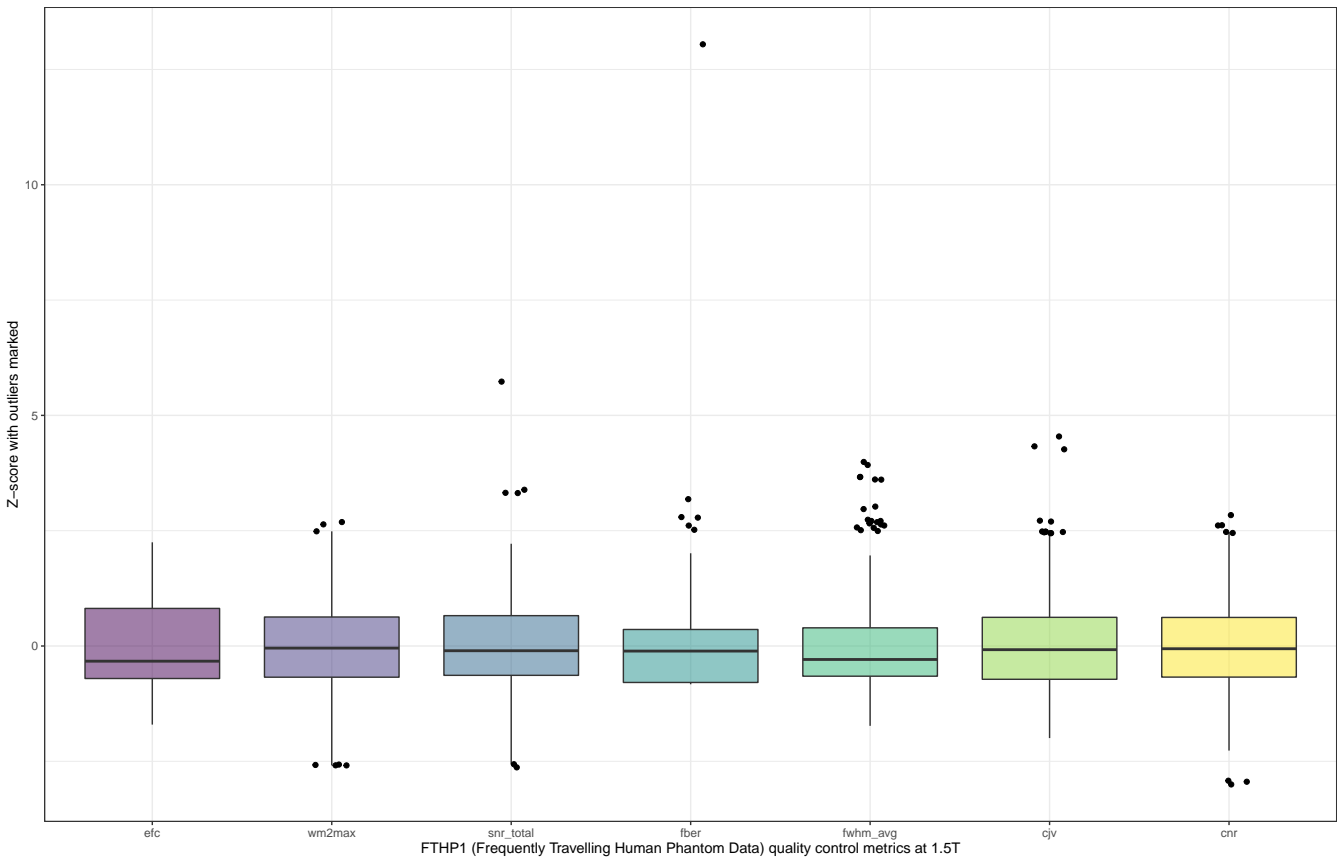
Only for FTHP1 data were collected at 1.5T, hence, qualuty control metrics are only present for this subject.

**Supplement 3:** Age, predicted age, brain age gap (BAG), and prediction error by cross-sectional data set and field strength

| **Dataset** | **Field Strength** | **N_subjects_** | **Mean Age** | **SD Age** | **Mean Prediction** | **SD Prediction** | **Mean BAG** | **SD BAG** | **MAE** | **RMSE** |
| --- | --- | --- | --- | --- | --- | --- | --- | --- | --- | --- |
| TOP GE750 | 3T | 543 | 34.15 | 11.54 | 31.37 | 11.11 | -2.78 | 4.10 | 3.74 | 4.96 |
| TOP HDxt | 3T | 313 | 30.81 | 8.15 | 29.65 | 8.76 | -1.16 | 3.78 | 3.05 | 3.95 |
| TOP all | 3T | 856 | 32.93 | 10.55 | 30.74 | 10.34 | -2.19 | 4.06 | 3.49 | 4.61 |
| NCNG | 1.5T | 209 | 54.66 | 14.50 | 56.02 | 14.50 | 1.36 | 3.28 | 2.83 | 3.55 |

The presented data refer to the cross-sectional data used as a comparison to the longitudinal data presented in **Table 1**. TOP 3T data were obtained at two scanners: GE750 and HDxt. BAG = brain age gap, MAE = mean absolute error, RMSE = root mean squared error. BAG is calculated as the difference between predicted age and age.
